# Potato Virus X Vector-Mediated DNA-Free Genome Editing in Plants

**DOI:** 10.1093/pcp/pcaa123

**Published:** 2020-09-29

**Authors:** Hirotaka Ariga, Seiichi Toki, Kazuhiro Ishibashi

**Affiliations:** 1 Plant and Microbial Research Unit, Division of Plant and Microbial Sciences, Institute of Agrobiological Sciences, National Agriculture and Food Research Organization, Tsukuba, 305-8602 Japan; 2 Plant Genome Engineering Research Unit, Division of Applied Genetics, Institute of Agrobiological Sciences, National Agriculture and Food Research Organization, Tsukuba, 305-8602 Japan; 3 Graduate School of Nanobioscience, Yokohama City University, Yokohama, 236-0027 Japan; 4 Kihara Institute for Biological Research, Yokohama City University, Yokohama, 236-0027 Japan; 5 Plant Diversity Research Team, Genetic Resources Center, National Agriculture and Food Research Organization, Tsukuba 305-8602, Japan

**Keywords:** CRISPR-Cas9, *Nicotiana benthamiana*, Plant genome editing, RNA virus, Virus vector

## Abstract

Genome editing technology is important for plant science and crop breeding. Genome-edited plants prepared using general CRISPR-Cas9 methods usually contain foreign DNA, which is problematic for the production of genome-edited transgene-free plants for vegetative propagation or highly heterozygous hybrid cultivars. Here, we describe a method for highly efficient targeted mutagenesis in *Nicotiana benthamiana* through the expression of Cas9 and single-guide (sg)RNA using a potato virus X (PVX) vector. Following *Agrobacterium*-mediated introduction of virus vector cDNA, >60% of shoots regenerated without antibiotic selection carried targeted mutations, while ≤18% of shoots contained T-DNA. The PVX vector was also used to express a base editor consisting of modified Cas9 fused with cytidine deaminase to introduce targeted nucleotide substitution in regenerated shoots. We also report exogenous DNA-free genome editing by mechanical inoculation of virions comprising the PVX vector expressing Cas9. This simple and efficient virus vector-mediated delivery of CRISPR-Cas9 could facilitate transgene-free gene editing in plants.

## Introduction

The CRISPR-Cas9 [clustered regularly interspaced short palindromic repeats (CRISPR)-associated protein 9] system is used for targeted mutagenesis in various plant species (Erpen-Dalla et�al. 2019, Manghwar et�al. 2019). In most cases, plant genome editing using CRISPR-Cas9 is achieved via *Agrobacterium*-mediated transformation of exogenous DNA encoding Cas9 and a single-guide RNA (sgRNA). After segregation, transformants carrying the targeted mutation are free from exogenous DNA in the next generation; however, exogenous DNA is difficult to remove from vegetatively propagated plants or highly heterozygous hybrid cultivars. For these and regulatory reasons, methods to generate genome-edited plants without integration of foreign DNA into their genomes have been developed. First, transfection of ribonucleoprotein (RNP; preassembled sgRNA and Cas9 protein complex) into protoplasts and subsequent regeneration of whole plants was used successfully to introduce mutations in several plant species (Woo et�al. 2015, Svitashev et�al. 2016, Andersson et�al. 2018). Second, targeted mutations were introduced into plants through biolistic bombardment, where gold particles carrying CRISPR-Cas9 components were fired into the shoot apical meristems of imbibed seeds (Liang et�al. 2017, Hamada et�al. 2018). Finally, shoot regeneration from tobacco leaves infected with *Agrobacterium* harboring CRISPR-Cas9 expression vectors in the absence of antibiotic selection resulted in the isolation of transgene-free mutants (Chen et�al. 2018). While these approaches are expected to be useful for plant genome editing, they have technical limitations, including the range of applicable plant species and low efficiency. In addition, plants regenerated from protoplasts often carry undesired somaclonal mutations.

Virus vectors have been used widely in plants for transient expression of foreign proteins and gene silencing. Viral vectors are amplified within the cells into which they are introduced, enabling high-level expression, and are promising tools for transgene integration-free genome editing (Cody and Scholthof 2019). Previous attempts to use plant RNA virus vectors, including tobacco mosaic virus, tobacco rattle virus, pea early browning virus, barley stripe mosaic virus, beet necrotic yellow vein virus and foxtail mosaic virus, for the expression of sgRNA were successful in introducing mutations into host genomes when Cas9 protein was supplied in trans (Cody et�al. 2017, Ali et�al. 2018, Hu et�al. 2019, Jiang et�al. 2019, Mei et�al. 2019, Ellison et�al. 2020). However, virus vector-mediated expression of Cas9 is challenging due to the large size of Cas9, as the length of the foreign gene insert correlates negatively with the stability of plant virus vectors (Avesani et�al. 2007). DNA replicons based on deconstructed geminiviruses have expressed Cas9 successfully (Baltes et�al. 2014, Čerm�k et�al. 2015, Gil-Humanes et�al. 2017). The insert size for geminivirus vectors is, however, physically restricted; the viral genomic DNA is packed into virion shells, and the deconstructed replicons lack movement protein (MP) and coat protein (CP) genes; due to this lack of MP and CP genes, they are not infectious on their own. By contrast, potato virus X (PVX), a frequently used plant RNA virus vector, has a filamentous flexible structure that consists of an RNA genome wrapped in ∼1300 units of a single CP (Kendall et�al. 2008). Thus, it is unlikely that gene insert size is physically limited in the PVX vector, making its use as an autonomously replicating vector for the delivery of large sequence-specific nuclease (SSN) genes feasible. Here, we aimed to establish an exogenous DNA-free plant genome editing method using the PVX vector to deliver both Cas9 and sgRNA.

## Results

### Vector construction for PVX-mediated genome editing

RNA virus vector-mediated plant genome editing has been hindered because large foreign nucleotide sequences in the virus genome are unstable and are typically deleted during replication. These deletion mutants outcompete the parental virus vector during replication and are rapidly selected for through cell-to-cell spread processes (Miyashita et�al. 2015). To minimize the number of replication and cell-to-cell movement cycles of virus vectors carrying the *Cas9* gene, we first used *Agrobacterium*-mediated introduction of viral cDNA into the cells of infiltrated leaves (agroinfection). The *SpCas9* gene and its cognate sgRNA sequence were inserted into PVX cDNA cloned into the binary vector pPZP2028 (Endo et�al. 2015). PVX genomic RNA is going to be transcribed from T-DNA by cellular RNA polymerase II in infected cells. The genomic RNA is served as a template for translation to produce the viral RNA polymerase by which genomic RNA is replicated and subgenomic RNA acting as an mRNA for SpCas9 is transcribed. The sgRNA sequence was inserted immediately downstream of the *SpCas9* stop codon without including RNA processing systems, such as ribozymes, as extra nucleotides added to the 5′ and 3′ ends of the sgRNA do not affect the targeted mutagenesis efficiency of SpCas9 in planta (Mikami et�al. 2017, Cody and Scholthof 2020). Possibly, SpCas9 binds the sgRNA sequence in viral genomic or subgenomic RNA and then extra nucleotide sequences are trimmed by cellular nucleases (Cody and Scholthof 2020). The plasmid DNA construct was named pPZPVX-Cas9, from which PVX-Cas9 (PVX vector containing *SpCas9* and sgRNA) is expressed in plant cells (Fig.�1A, Supplementary Fig. S1).

**Fig. 1:**
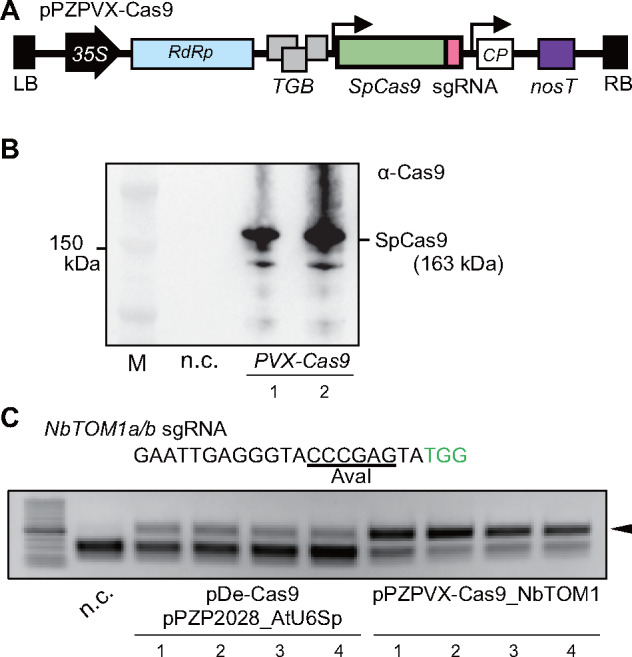
Targeted mutagenesis by agroinoculation with PVX vector expressing Cas9 and sgRNA. (A) Schematic diagram of a PVX-based SpCas9 expression vector. A full-length *SpCas9* sequence was placed after a duplicated subgenomic promoter for coat protein (shown by arrows). The sgRNA was fused to the 3′ end of the *SpCas9* coding sequence*. 35S*, Cauliflower mosaic virus 35S RNA promoter; *RdRp*, RNA-dependent RNA polymerase; *TGB*, triple gene block; *CP*, coat protein; *nosT*, *nopaline synthase* terminator; LB, left border of T-DNA; RB, right border of T-DNA. (B) Western blot analysis of SpCas9 accumulation in PVX-Cas9-inoculated *Nicotiana benthamiana* leaves at 5 dai. Lanes 1 and 2 are independent plants. Lane M, protein size markers; n.c., non-inoculated control *N.�benthamiana* leaf. (C) CAPS analysis for the detection of introduced mutations in agroinfiltrated *N.�benthamiana* leaves. Mixtures of *Agrobacterium* strains harboring pRI-p19, pDe-Cas9 and pPZP2028_AtU6Sp_NbTOM1, or pRI-p19 and pPZPVX-Cas9_NbTOM1 were infiltrated and DNA was extracted from four independent plants at 7�dai. PCR products containing the target site in *NbTOM1a/b* genes were digested with AvaI. Black triangle indicates undigested bands. Green letters indicate the PAM sequence. n.c., nontreatment control.

### PVX-Cas9-mediated targeted mutagenesis in *Nicotiana benthamiana*

We examined whether PVX-Cas9 introduced targeted mutations in agroinfiltrated *Nicotiana benthamiana* leaves. sgRNAs for *NbTOM1* or *NbPDS* corresponding to the endogenous *TOBAMOVIRUS MULTIPLICATION 1* (*TOM1*) and *PHYTOENE DESATURASE* (*PDS*) genes, respectively, were designed. Since *N.�benthamiana* is an allotetraploid plant, two homologous genes for *TOM1* (*NbTOM1a* and *NbTOM1b*) and *PDS* (*NbPDSa* and *NbPDSb*) are found in the *N.�benthamiana* genome. The sgRNA for *NbTOM1* targeted identical regions of *NbTOM1a* and *NbTOM1b* (Supplementary Fig. S2). A previously reported sgRNA for the *Nicotiana tabacum PDS* gene (Kaya et�al. 2016) was used for *NbPDS*, which targeted *NbPDSa* but had a 1-base mismatch for *NbPDSb* (Supplementary Fig. S2). *Agrobacterium* harboring pPZPVX-Cas9_NbTOM1 or pPZPVX-Cas9_NbPDS was infiltrated into leaves of *N.�benthamiana* along with *Agrobacterium* harboring the pRI-p19 plasmid expressing the p19 protein—an RNA silencing suppressor derived from tomato bushy stunt virus. In *N.�benthamiana* leaves infiltrated with pPZPVX-Cas9_NbTOM1, full-length SpCas9 protein of the predicted molecular size (163�kDa) was detected by Western blot analysis at 5 d after inoculation (dai; Fig.�1B). At 7 dai, DNA extracted from the infiltrated leaves was subjected to cleaved amplified polymorphic sequence (CAPS) analysis to determine whether mutations were introduced into the targeted sequences. In all samples from individual plant leaves infiltrated with pPZPVX-Cas9_NbTOM1 or pPZPVX-Cas9_NbPDS, undigested bands representing mutation(s) introduced into the target restriction enzyme sites were detected (Fig.�1C, Supplementary Fig. S3). The genome editing efficiency in PVX-Cas9-inoculated leaves was markedly higher than in agroinfiltrated leaves that expressed SpCas9 and sgRNA transiently from the ubiquitin (pDe-Cas9; Fauser et�al. 2014) and U6 snRNA (pPZP2028_AtU6Sp_NbTOM1) promoters, respectively, with p19 (Fig.�1C).

The improved genome editing efficiency in PVX-Cas9-inoculated leaves prompted us to try to obtain marker-free genome-edited plants by the regeneration of shoots in the absence of antibiotic selection. Explants prepared from pPZPVX-Cas9_NbTOM1 agroinoculated, surface-sterilized leaves at 7�dai were placed onto callus and shoot induction medium (Fig.�2A). Cefotaxime was added to the medium to kill *Agrobacterium*, but no antibiotics for the selection of transformants were added. DNA was extracted from the leaves of regenerated shoots, and mutations in the target site within the *NbTOM1a/b* genes were detected by CAPS analysis. Among 50 regenerated shoots from three independent experiments, more than half (31/50) carried mutations in either *NbTOM1a* or *NbTOM1b*, or both (Fig.�2B, C). Sequence analysis revealed that regenerated shoot # 1 carried heterozygous mutations in both *NbTOM1a* (none/1-bp substitution) and *NbTOM1b* (none/7-bp deletion), whereas regenerated shoot # 10 had biallelic mutations in both *NbTOM1a* (1-bp insertion/2-bp deletion) and *NbTOM1b* (8-bp deletion/8-bp deletion and 1-bp substitution) (Fig.�2D). In our selection-free method, PCR detected pPZPVX-Cas9_NbTOM1-derived DNA in 18% (9/50) of the regenerated shoots (Fig.�2B, C). By contrast, PVX RNA was detected in 88% (15/17) of the shoots by RT-PCR (Fig.�2B). Thus, PVX-Cas9 RNA infected most cells of the infiltrated leaves and had replicated to express a large amount of Cas9 protein, while T-DNA integration into *N. benthamiana* genomes occurred at a lower frequency. On the other hand, only 1.56% (1/64) of the shoots that were regenerated without an antibiotic selection from leaf explants that had been infiltrated with *Agrobacterium* harboring the pRI-p19, pDe-Cas9 and pPZP2028_AtU6Sp_NbTOM1 plasmids carried mutations (Supplementary Table S2). This improvement in genome editing efficiency using PVX-Cas9 is a considerable advancement toward DNA integration-free genome editing by allowing regeneration without selection.

**Fig. 2:**
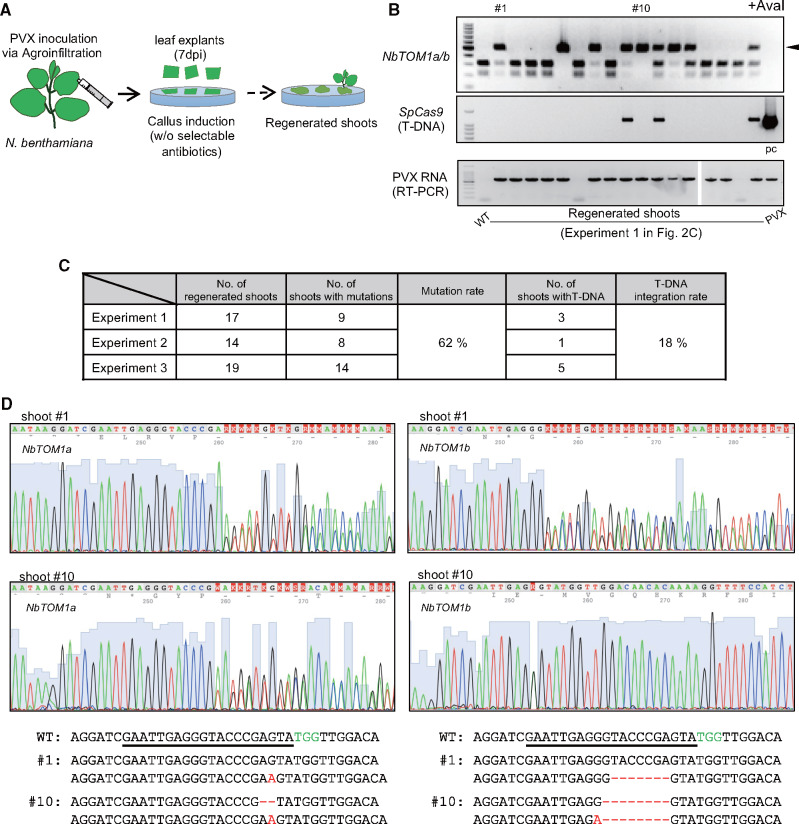
Transgene integration-free genome editing using PVX vector through regeneration without selection. (A) Flowchart for PVX-mediated genome editing of *N.�benthamiana*. Throughout the process, no antibiotics for the selection of genome-edited cells were added to the medium. (B) Characterization of regenerated shoots. Mutations in the target site within the *NbTOM1a/b* gene were detected by CAPS analysis. PCR products were digested with AvaI (top). Presence of T-DNA derived from pPZPVX-Cas9_NbTOM1 was detected by PCR (middle). PVX RNA was detected by RT-PCR (bottom). WT, untreated *N. benthamiana*; pc, pPZPVX-Cas9 plasmid DNA was used as a PCR template for positive control; PVX, RNA extracted from PVX-infected *N. benthamiana* was used as a template for RT-PCR. (C) Summary of the mutation and T-DNA integration rates in shoots regenerated from PVX-Cas9-inoculated leaves from three independent experiments. These rates were calculated from the sum of three independent experiments. (D) DNA sequences around the target region in *NbTOM1a/b* in regenerated shoots. WT, wild-type sequence. Underlined bases indicate the target sequence and green letters indicate the PAM sequence. Red letters represent DNA insertion or substitution, and dashes indicate deletion.

To examine whether the mutation induced by PVX-Cas9 was inherited, DNA was extracted from leaves of progeny plants of the regenerated shoots # 1 and # 10. DNA fragments encompassing the mutation sites of *NbTOM1a* and *NbTOM1b* were amplified independently using specific primers for CAPS analysis. In the progeny of shoot # 1, which had heterozygous mutations in both *NbTOM1a* and *NbTOM1b*, mutant alleles segregated (Supplementary Fig. S4A, left panel). On the other hand, all individual progeny of shoot # 10 were homozygous for mutant alleles in both *NbTOM1a* and *NbTOM1b* (Supplementary Fig. S4A, right panel). These results indicate that the mutation induced by PVX-Cas9 was inherited in the next generation. To determine whether PVX RNA could be transmitted through seed to the next generation, PVX genomic RNA levels in the leaves of the progeny derived from shoot # 1 and shoot # 10 were assessed by RT-PCR. PVX RNA levels were below the detection limit of RT-PCR in all plants tested (Supplementary Fig. S4B), suggesting that transgenerational transmission of PVX is unlikely to occur. We therefore obtained genome-edited plants that potentially contain neither exogenous DNA nor viral RNA.

### PVX vector-mediated C-to-T base editing

Base editing enables targeted nucleotide substitutions, which expands the possibilities of genome editing. Base editors consisting of SpCas9 fused to nucleoside deaminase (Komor et�al. 2016, Nishida et�al. 2016, Gaudelli et�al. 2017) are inevitably larger than SpCas9 itself. To ascertain whether the PVX vector could be used for base editing, the SpCas9 coding sequence in pPZPVX-Cas9 was replaced with that of nSpCas9-NGv1-AID (Endo et�al. 2019), a fusion protein consisting of cytidine deaminase and nickase SpCas9 (nSpCas9) with a protospacer adjacent motif (PAM) sequence modified to recognize NG to produce the plasmid pPZPVX-AID (Fig.�3A, Supplementary Fig. S1). The sgRNA was redesigned to contain putative target cytosine residues in an AvaI restriction site (CYCGRG) in the *NbTOM1a/b* gene (Supplementary Fig. S2). Shoots were induced from explants derived from pPZPVX-AID_NbTOM1 agroinoculated *N. benthamiana* leaves at 7 dai without antibiotic selection. CAPS analysis revealed that 61% (27/44) of the shoots carried mutations (Fig.�3B, C). Sequence analysis of the representative shoots confirmed that the introduced mutations were indeed C-to-T substitutions at the nucleoside 17 bases upstream of the PAM (Fig.�3D). Thus, the PVX vector system is compatible with a base editing for integration-free targeted nucleotide changes.

**Fig. 3:**
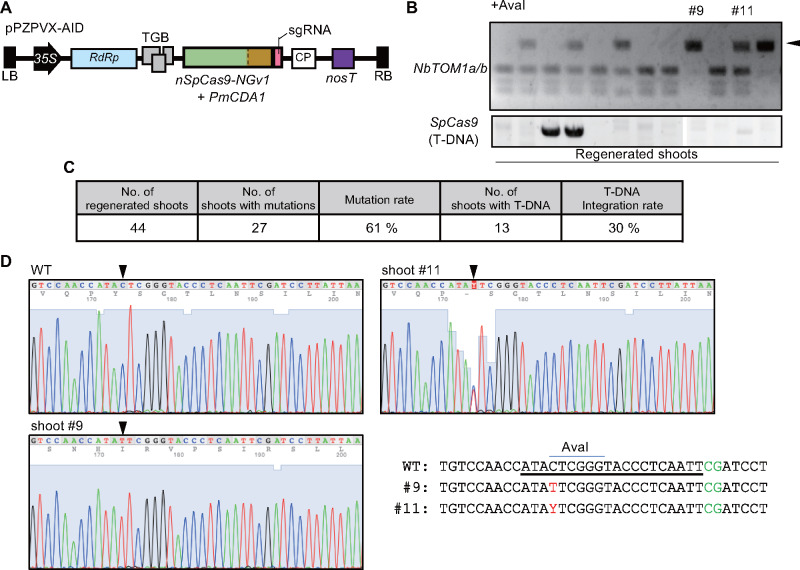
C-to-T base editing using a PVX vector. Schematic diagram of pPZPVX-AID. *35S*, *Cauliflower mosaic virus 35S* promoter; *RdRp*, RNA-dependent RNA polymerase; *TGB*, Triple gene block; *CP*, coat protein; *nosT*, *nopaline synthase* terminator; *PmCDA1*, *Petromyzon marinus* cytidine deaminase 1; LB, left border of T-DNA; RB, right border of T-DNA. (B) Detection of mutations in regenerated shoots by CAPS analysis. Shoots were regenerated from pPZPVX-AID_NbTOM1-inoculated *N. benthamiana* leaves as in Fig.�2A. PCR products encompassing the target site in the *NbTOM1a/b* gene were digested with *Ava*I (upper panel). Presence of T-DNA derived from pPZPVX-AID_NbTOM1 was detected by PCR (lower panel). Black triangle indicates undigested bands. (C) Summary of the mutation and T-DNA integration rates of shoots regenerated from pPZPVX-AID-inoculated leaves. (D) DNA sequences around the target region of *NbTOM1a/b* (analyzed collectively) in regenerated shoots. WT, wild-type sequence. Underlined bases indicate the target sequence, and green letters indicate the PAM sequence. Red letters represent nucleotide substitutions.

### DNA-free targeted mutagenesis by mechanical inoculation of PVX-Cas9

Although DNA integration-free genome-edited plants were obtained at high frequency by agroinfection with the PVX vector, this method permits the transient introduction of foreign DNA into plant cells. To avoid the possibility of integration of any small DNA fragments, we attempted exogenous DNA-free genome editing by mechanical inoculation, i.e. rub inoculation of virions of PVX-Cas9 comprising genomic RNA and CP with abrasive carborundum into leaves. To prepare an inoculum, *Agrobacterium* harboring pPZPVX-Cas9_NbTOM1 was first infiltrated into *N.�benthamiana* leaves as above, and the leaves were homogenized at 7 dai followed by filter sterilization. The filtered sap was mechanically inoculated onto new *N. benthamiana* leaves, and then, shoots were regenerated from the inoculated leaves without antibiotic selection (Fig.�4A). The targeted mutations were detected in the inoculated leaves by CAPS analysis (Fig.�4B). Among the regenerated shoots, genome-edited shoots were found with biallelic mutations in both *NbTOM1a* and *NbTOM1b*, although the efficiency was lower than that with agroinoculation (Fig.�4C–E, Supplementary Fig. S5). Thus, we successfully edited a plant genome without introducing exogenous DNA by mechanical inoculation of the PVX-Cas9 RNA virus vector.

**Fig. 4:**
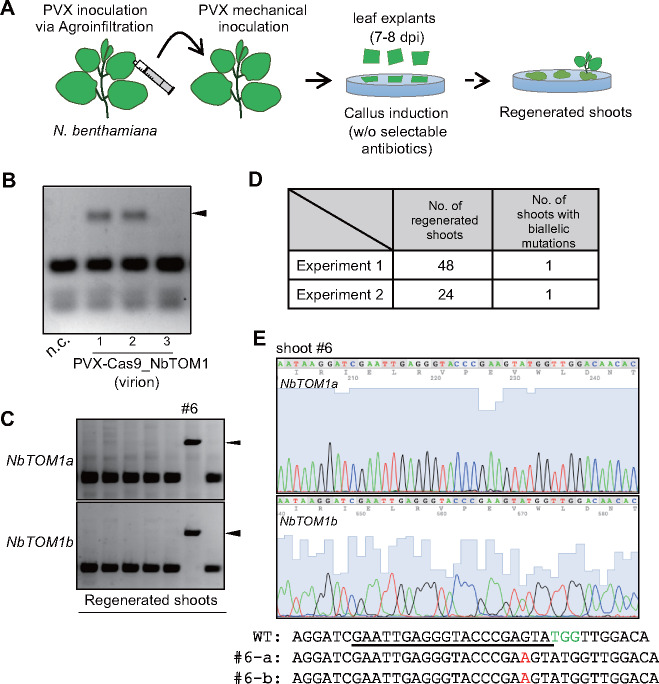
DNA-free genome editing using a PVX vector. (A) Flowchart for PVX-mediated DNA-free genome editing of *N.�benthamiana*. Filter-sterilized leaf sap from PVX-Cas9 agroinfiltrated leaves was used for mechanical inoculation. Throughout the process, no antibiotics for the selection of genome-edited cells were added to the medium. (B) CAPS analysis for the detection of introduced mutations in mechanically inoculated *N.�benthamiana* leaves. DNA was extracted from three independent plants at 18�dai. PCR products containing the target site in *NbTOM1a/b* genes were digested with AvaI. Black triangle indicates undigested bands. (C) Characterization of regenerated shoots. Mutations in the target site within the *NbTOM1a* and *NbTOM1b* genes were independently detected by CAPS analysis. PCR products were digested with AvaI. (D) Summary of the mutation rates in shoots regenerated from PVX-Cas9-inoculated leaves in two independent experiments. (E) DNA sequences around the target region.

## Discussion

SSN delivery systems that do not rely on genomic integration or the introduction of foreign DNA are in high demand, particularly for editing the genomes of crops for which null segregants are difficult to obtain and when non-transgenic progeny are desired for technical or regulatory reasons. Virus vectors are promising tools for non-transgenic genome editing, while the large size of SSNs has been a technical hurdle. In this study, we described methods for PVX vector-mediated genome editing. In the agroinfection system, >60% of shoots regenerated from agroinfiltrated leaves under nonselective conditions had targeted mutations, while exogenous DNA derived from pPZPVX-Cas9, the plasmid carried by the infiltrated *Agrobacterium*, was detected by PCR in only 18% (9/50) of regenerated shoots (Fig.�2B, C). This result suggests that T-DNA integration is a relatively rare event and that transgene-free genome-edited plants could be readily obtained. By contrast, PVX RNA was detected in 88% of the regenerated shoots (Fig.�2B). We did not observe vertical transmission of PVX and obtained virus-free genome-edited plants through seed propagation. Residual virus RNA in regenerated shoots can be a problem in vegetatively propagated plants because there are no antiviral drugs that can eliminate plant viruses from infected plants. Previously, we developed a CP-deficient tomato mosaic virus vector that carries a target site for tobacco miroRNA398 (miR398), expression of which is strongly induced when leaf explants are placed on callus induction medium, resulting in the elimination of viral RNA in regenerated tissues (Chujo et�al. 2017). We inserted the miR398 target sequence into the PVX genome; however, PVX RNA remained in regenerated shoots (data not shown) probably because the encapsidated PVX genomic RNA cannot be accessed by miR398. Although PVX CP is required for cell-to-cell movement of the virus, deletion of the CP gene, or replacement of CP with other viral MPs to complement the movement function, may satisfy requirements for a PVX-based eliminable virus vector for agroinfection. Otherwise, traditional shoot-tip culture may be a good alternative for obtaining virus-free clones for vegetatively propagated plants.

For practical use of genome editing, base editors are useful tools for targeted nucleotide changes, which are fusion proteins of Cas9 and base-modifying enzymes, such as cytidine deaminase or adenosine deaminase (Komor et�al. 2016, Nishida et�al. 2016, Gaudelli et�al. 2017). However, the size of base editors is properly larger than Cas9, which made it difficult to deliver them into cells by virus vectors. PVX-AID carrying the cytidine deaminase-fused SpCas9 (1813 aa) successfully induced C-to-T substitution in regenerated shoots from agroinoculated leaves (Fig.�3). This result highlights the merit of the PVX system in transgene integration-free base editing of plants. Because PVX mainly infects Solanaceae plants, including potato, tomato, eggplant and pepper, PVX-mediated targeted mutagenesis would be applicable to these commercially valuable crops.

Very recently, technical breakthroughs in delivering CRISPR-Cas9 components into plant cells using virus vectors have been reported. A negative-strand RNA virus vector based on Sonchus yellow net rhabdovirus (SYNV) was used successfully to express SpCas9 and sgRNA to achieve DNA-free genome editing (Ma et�al. 2020). A major difference between positive-strand RNA viruses, including PVX, and negative-strand RNA viruses like SYNV is that the genome RNAs of negative-strand RNA viruses are always wrapped with nucleocapsid proteins, whereas those of positive-strand RNA viruses are not. The PVX system would therefore be advantageous for inserting functional RNA elements, such as recently reported mobile elements, for the introduction of a positive-strand RNA virus vector into a shoot apical meristem (Ellison et�al. 2020). Generally, shoot apical meristems are protected from plant virus invasion by, at least in part, an antiviral RNA silencing system (Schwach et�al. 2005), which prevents heritable mutagenesis by virus vectors without tissue culture. Although it is currently difficult to express the SpCas9 protein in systemic tissues using the PVX vector system due to gene instability, plant genome editing may no longer require tissue culture if virus vectors expressing SSNs are delivered directly into meristems. A recently reported compact SSN, CasΦ (∼800 aa) from huge phages (Pausch et�al. 2020), may be a good alternative for SpCas9 for this purpose.

## Materials and Methods

### Construction of PVX-based genome editing vector

To generate a PVX vector expressing Cas9 and sgRNA, four unique restriction sites, SrfI, PvuI, BsaAI and MluI, were inserted between the NruI and SalI sites in pPVX201 (Baulcombe et�al. 1995). The cauliflower mosaic virus 35S promoter, the modified full-length PVX cDNA and nopaline synthase terminator sequences were ligated between the AscI and PmeI sites of pPZP2028 (Endo et�al. 2015) to produce the new vector pPZPVX301. The full-length *Cas9* gene from *Streptococcus pyogenes* (*SpCas9*), which was optimized for expression in plants and fused with a Simian virus 40 nuclear localization signal, was amplified from pDe-Cas9 (Fauser et�al. 2014) and inserted into the SrfI and MluI sites of pPZPVX301. sgRNA sequences comprising 80-bp scaffold RNA and guide RNAs for *NbTOM1* (GAATTGAGGGTACCCGAGTA) or *NbPDS* (TGCGATGCCTAACAAGCCAG) were ligated between the MluI and SalI sites, which immediately follow the *SpCas9* stop codon. The constructed PVX vector containing *SpCas9* and sgRNA was named pPZPVX-Cas9. For C-to-T base editing, the *SpCas9* sequence in the pPZPVX-Cas9 vector was replaced with the *nSpCas9-NGv1-AID* sequence (Endo et�al. 2019), which encodes a fusion protein containing cytidine deaminase (*Petromyzon marinus* cytidine deaminase 1) and nickase SpCas9 (nSpCas9) with a PAM sequence modified for NG to produce the pPZPVX-AID plasmid. A guide RNA was designed to introduce a synonymous substitution in the *NbTOM1* coding sequence (ATACTCGGGTACCCTCAATT), which was expected to have neutral effects on the growth of regenerated shoots. To express sgRNA from the AtU6-26 promoter from *Agrobacterium*-mediated transient expression, an sgRNA cassette for *NbTOM1* was cloned into pUC-AtU6-sgRNA (Endo et�al. 2019) and, then, the cassette was transferred into pPZP2028 (named pPZP2028_AtU6Sp_NbTOM1). Plasmid DNA was transfected into *Agrobacterium tumefaciens* C58C1 by electroporation. DNA sequences of pPZPVX-Cas9_NbTOM1 and pPZPVX-AID_NbTOM1 have been deposited to DNA Data Bank of Japan (DDBJ) with the accession number LC577760 and LC577761, and are shown in Supplementary Figs. S6 and S7, respectively.

### Plant growth conditions and shoot regeneration

*Nicotiana benthamiana* plants were grown on soil in a growth incubator under an 16-h light/8-h dark cycle at 25�C. Transient Cas9 and sgRNA expression from T-DNA and from the PVX vector was achieved via agroinfiltration as described previously (Kaya et�al. 2017). In brief, *A. tumefaciens* strains were cultured in Luria broth medium and suspended in infiltration buffer (10 mM 2-Morpholinoethanesulfonic acid (MES), pH 5.8, 10 mM MgCl_2_, 100 μg ml^−1^ acetosyringone). *Agrobacterium* expressing the p19 gene, which encodes the 19-kDa protein derived from *Tomato bushy stunt virus*, which enhances transient transgene expression, was co-infiltrated. PVX-inoculated leaves were collected 7 dai, treated with 1% NaOCl solution and, then, washed three times with sterilized water. The leaves were cut into ca. 0.8-cm squares to prepare explants and then placed on shoot induction medium (Murashige–Skoog basal salts, 2% sucrose, 0.1�mg l^–1^ 1-naphthaleneacetic acid, 1�mg l^–1^ 6-benzylaminopurine, 200�mg l^–1^ cefotaxime and 0.6% agar). Cefotaxime was replaced with carbenicillin (250�mg l^–1^) in some cases. Regenerated shoots were transferred to MS medium (Murashige–Skoog basal salts, 1% sucrose, 0.05% MES, vitamin mix and 0.8% agar) to induce rooting.

### Mechanical inoculation of PVX vector

*Agrobacterium* harboring the PVX-Cas9 expression plasmid was infiltrated into *N.�benthamiana* leaves, and the inoculated leaves were homogenized at 7�dai in 10�mM NaPi buffer (pH�7.0). The crude extract was clarified by centrifugation at 16,000�*g* for 3�min, followed by filtration through a 0.45-μm polyvinylidene fluoride filter. The clarified leaf sap and abrasive carborundum (600 mesh; Nacalai Tesque) were applied onto the fourth or fifth true leaves. The leaves were then rubbed gently by hand to inoculate PVX mechanically. Inoculated plants were grown at 16�C under an 16-h light/8-h dark cycle. Regeneration of shoots from the inoculated leaves was initiated at 7–8�dai as described above.

### Western blot analysis

Total protein was extracted from PVX-Cas9-inoculated leaves at 5�dai by grinding in extraction buffer [50�mM Tris-HCl pH�6.8, 2% (w/v) SDS, 10% (v/v) glycerol and 0.1�M β-mercaptoethanol]. Cas9 protein was detected using anti-Cas9 antibody (7A9-3A3; Active Motif, 1914 Palomar Oaks Way STE 150, Carlsbad, CA 92008, USA).

### Characterization of regenerated shoots

DNA for CAPS analysis and sequencing was extracted from regenerated shoots using the DNeasy Plant Mini Kit (QIAGEN, QIAGEN Strasse 1, 40724 Hilden, Germany) or DNAiso (TaKaRa, Nojihigashi 7-4-38, Kusatsu, Shiga 525-0058, Japan). PCR was performed with primers listed in Supplementary Table S1, and PCR products were digested with AvaI (for *NbTOM1a/b*) or BstNI (for *NbPDSa*) (New England Biolabs, 240 County Rd, Ipswich, MA 01938, USA). RNA was extracted from leaves of regenerated shoots using the RNeasy Plant Mini Kit (QIAGEN). PVX RNA was detected from 100 ng of total RNA by RT-PCR using the PrimeScript One-Step RT-PCR Kit (TaKaRa) for 40 reaction cycles.

## Supplementary Data

Supplementary data are available at PCP online.

## Funding

The Cabinet Office, Government of Japan, Cross-ministerial Strategic Innovation Promotion Program (SIP) ‘‘Technologies for Creating Next-generation Agriculture, Forestry, and Fisheries” and ‘‘Technologies for Smart Bio-industry and Agriculture” (funding agency: Bio-oriented Technology Research Advancement Institution, NARO) to K.I. and S.T.

## Supplementary Material

pcaa123_Supplementary_Data
