# Supplementary material for: Potato Virus X Vector-Mediated DNA-Free Genome Editing in Plants
Source: Plant Cell Physiol. 2020 Sep 29;61(11):1946–53. doi: 10.1093/pcp/pcaa123 (PMC7758033; doi:10.1093/pcp/pcaa123)
Supplement: pcaa123_Supplementary_Data [file pcaa123_supplementary_data.docx]

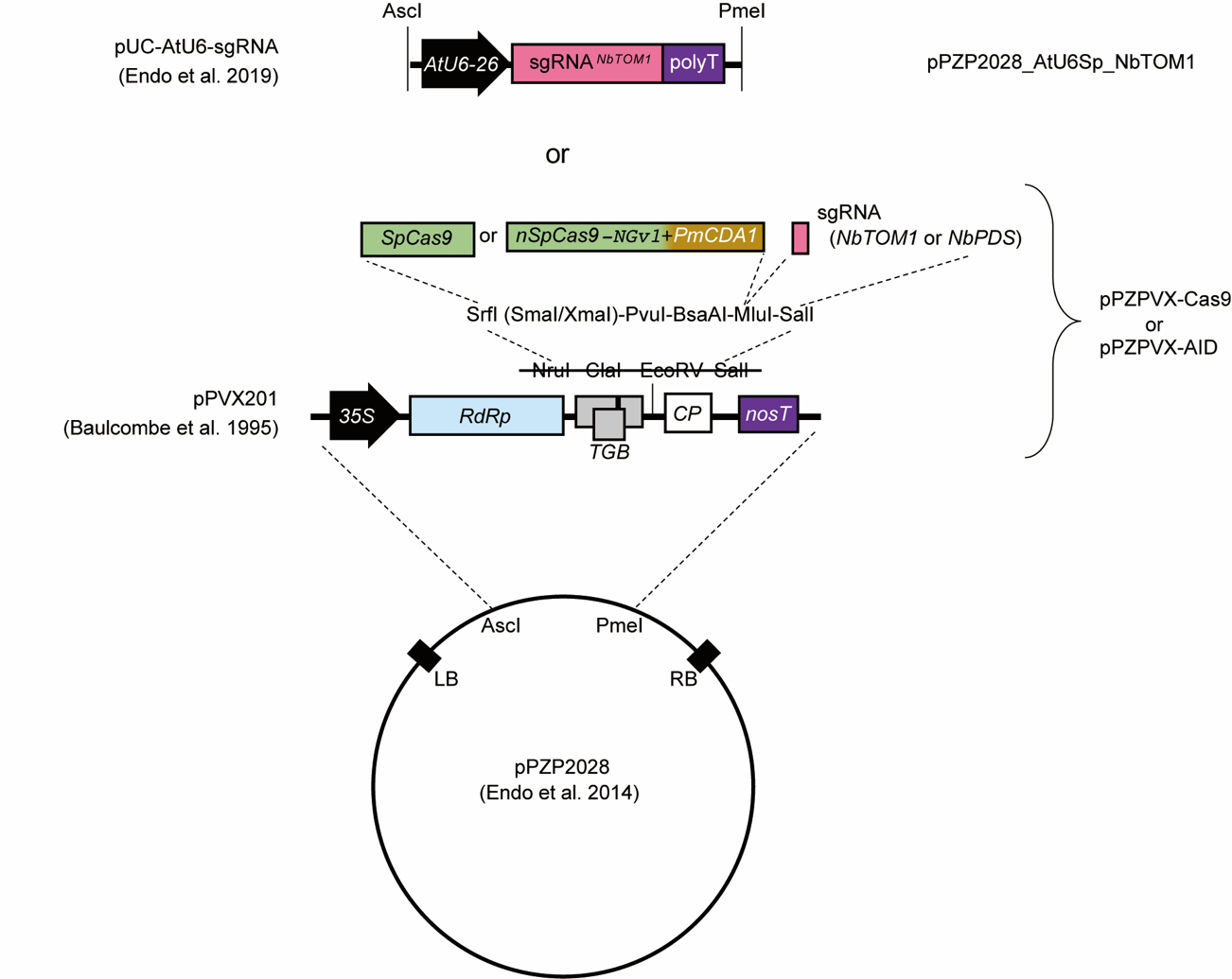


**Fig. S1 | Schematic diagrams of the plasmids used in this study.**

During replication of the PVX genome RNA, subgenomic RNA that acts as a messenger RNA for *SpCas9* or *nSpCas9-NGv1+PmCDA1* is transcribed. The sgRNA sequence is contained in both genomic and subgenomic RNAs which could be bound by SpCas9 followed by trimming of extra sequences by cellular nucleases. See Materials and Methods for details of vector construction**.**

**
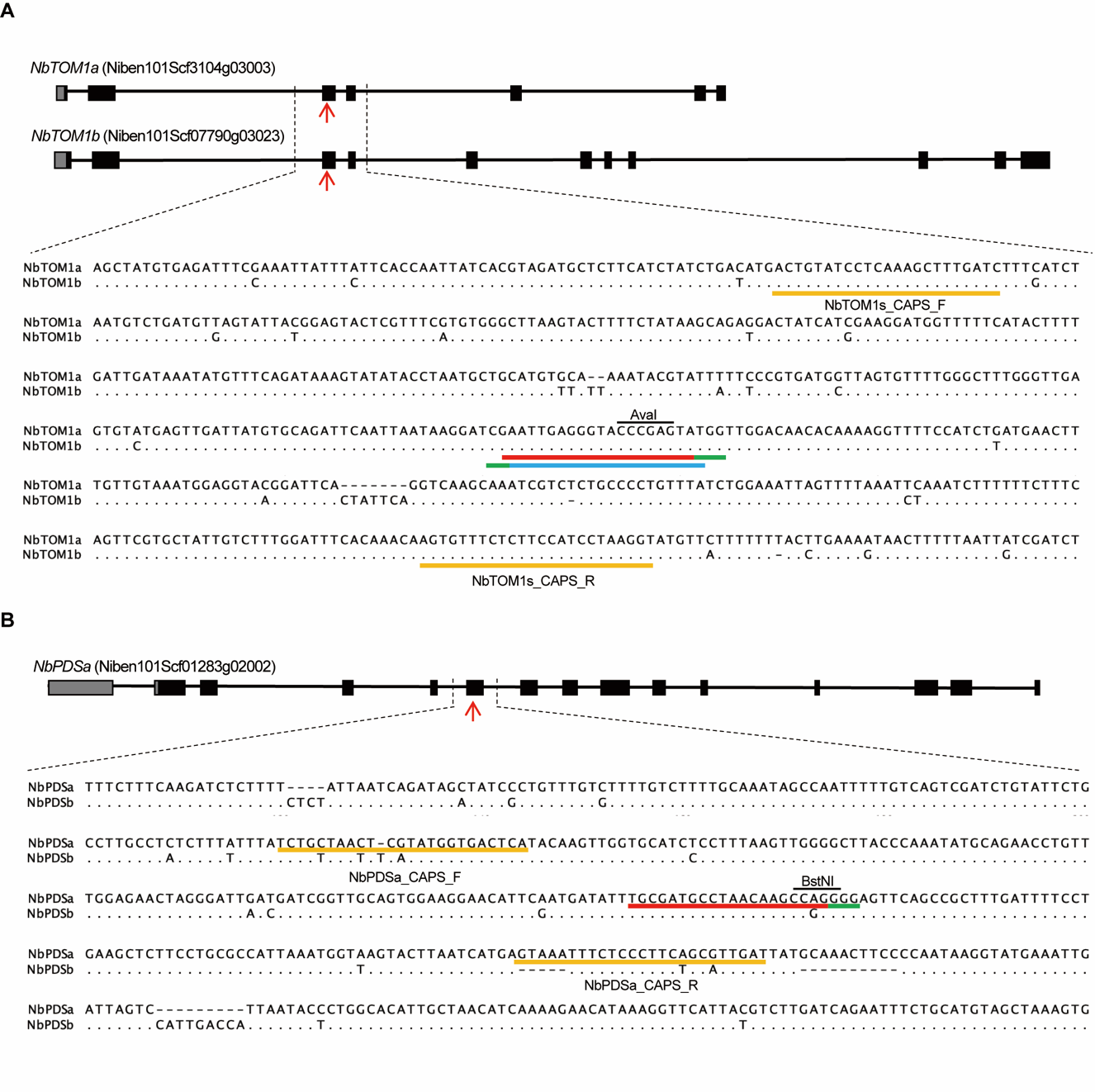
**

**Fig. S2 | Genomic sequences around the sgRNA target sites.**

Schematic diagrams and alignment of the *NbTOM1a* and *NbTOM1b* genes (A) and a diagram of the *NbPDSa* gene and alignment of the *NbPDSa* and *NbPDSb* sequences (B). Black boxes show exons and dark gray boxes show the 5′ UTR. Identical nucleotides and indels are indicated as dots and dashes, respectively. Red and cyan lines show the sgRNA target sites used by PVX-Cas9 and PVX-AID, respectively. Green lines show the PAM sequences. Positions of the primers used for CAPS analyses are marked by orange lines.


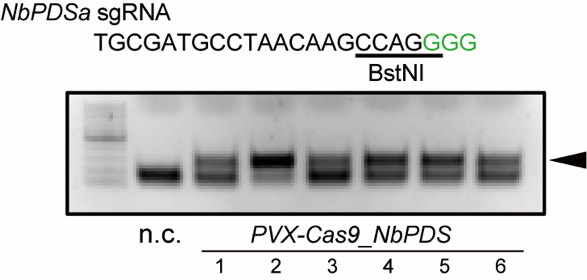


**Fig. S3 | PVX-Cas9-introduced targeted mutations in the *NbPDSa* gene.**

A mixture of *Agrobacterium* harboring pRI-p19 and pPZPVX-Cas9_NbPDS was infiltrated into *N. benthamiana* leaves and DNA was extracted at 7 dai. PCR products containing the target site were digested with BstNI. Lanes represent individual plants. Black triangle indicates undigested bands. Green letters indicate the PAM sequence. n.c., non-treated control.

**Fig. S4 | Virus-free genome-edited plants obtained by seed propagation.**

(A) Segregation patterns of the introduced mutations in the progeny of shoot #1 and shoot #10. Genomic DNA sequences around the target site in *NbTOM1a* and *NbTOM1b* were separately amplified followed by digestion with AvaI to detect the presence of the mutation. Lanes represent independent plants. (B) Absence of PVX RNA in the progeny of shoot #1 and #10. RT-PCR was performed as in Figure 2B for 40 cycles.


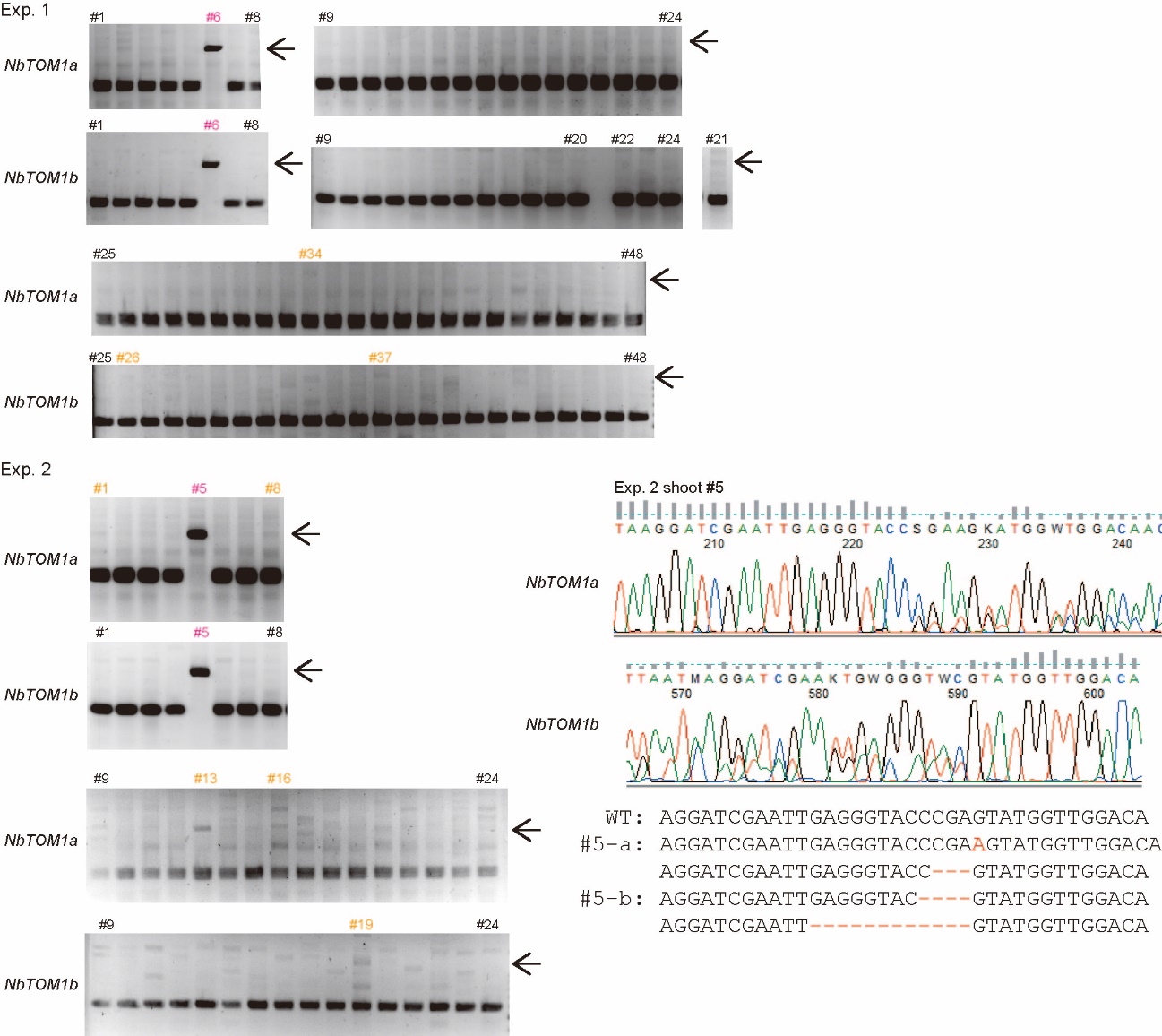


**Fig. S5 | CAPS analysis of regenerated shoots from leaves mechanically inoculated with PVX-Cas9_NbTOM1.**

DNA fragments encompassing the sgRNA target site of *NbTOM1a* and *NbTOM1b* were independently amplified and cleaved by AvaI. Shoots with biallelic mutations are indicated by magenta numbers. Undigested bands were faintly observed and are indicated by orange numbers. Electropherogram shows the DNA sequences around the target regions of *NbTOM1a* and *NbTOM1b* in regenerated shoot #5 from Exp. 2.

>pPZPVX-Cas9_NbTOM1

LB

tggcaggatatattgtggtgtaaacaaattgacgcttagacaacttaataacacattgcggacgtttttaatgtactgaattaacgccgaattgctctagcattcgccattcaggctgcgcaactgttgggaagggcgatcggtgcgggcctcttcgctattacgccagctggcgaaagggggatgtgctgcaaggcgattaagttgggtaacgccagggttttcccagtcacgacgttgtaaaacgacggccagtgccaagctggcgcgtctcagaagaccaaagggcaatTGAGACTTTTCAACAAAGGGTAATATCCGGAAACCTCCTCGGATTCCATTGCCCAGCTATCTGTCACTTTATTGTGAAGATAGTGGAAAAGGAAGGTGGCTCCTACAAATGCCATCATTGCGATAAAGGAAAGGCCATCGTTGAAGATGCCTCTGCCGACAGTGGTCCCAAAGATGGACCCCCACCCACGAGGAGCATCGTGGAAAAAGAAGACGTTCCAACCACGTCTTCAAAGCAAGTGGATTGATGTGATATCTCCACTGACGTAAGGGATGACGCACAATCCCACTATCCTTCGCAAGACCCTTCCTCTATATAAGGAAGTTCATTTCATTTGGAGAGGAgaaaactaaaccatacaccaccaacacaaccaaacccaccacgcccaattgttacacacccgcttgaaaaagaaagtttaacaaatggccaaggtgcgcgaggtttaccaatcttttacagactccaccacaaaaactctcatccaagatgaggcttatagaaacattcgccccatcatggaaaaacacaaactagctaacccttacgctcaaacggttgaagcggctaatgatctagaggggttcggcatagccaccaatccctatagcattgaattgcatacacatgcagccgctaagaccatagagaataaacttctagaggtgcttggttccatcctaccacaagaacctgttacatttatgtttcttaaacccagaaagctaaactacatgagaagaaacccgcggatcaaggacattttccaaaatgttgccattgaaccaagagacgtagccaggtaccccaaggaaacaataattgacaaactcacagagatcacaacggaaacagcatacattagtgacactctgcacttcttggatccgagctacatagtggagacattccaaaactgcccaaaattgcaaacattgtatgcgaccttagttctccccgttgaggcagcctttaaaatggaaagcactcacccgaacatatacagcctcaaatacttcggagatggtttccagtatataccaggcaaccatggtggcggggcataccatcatgaattcgctcatctacaatggctcaaagtgggaaagatcaagtggagggaccccaaggatagctttctcggacatctcaattacacgactgagcaggttgagatgcacacagtgacagtacagttgcaggaatcgttcgcggcaaaccacttgtactgcatcaggagaggagacttgctcacaccggaggtgcgcactttcggccaacctgacaggtacgtgattccaccacagatcttcctcccaaaagttcacaactgcaagaagccgattctcaagaaaactatgatgcagctcttcttgtatgttaggacagtcaaggtcgcaaaaaattgtgacatttttgccaaagtcagacaattaattaaatcatctgacttggacaaatactctgctgtggaactggtttacttagtaagctacatggagttccttgccgatttacaagctaccacctgcttctcagacacactttctggtggcttgctaacaaagacccttgcaccggtgagggcttggatacaagagaaaaagatgcagctgtttggtcttgaggactacgcgaagttagtcaaagcagttgatttccacccggtggatttttctttcaaagtggaaacttgggacttcagattccaccccttgcaagcgtggaaagccttccgaccaagggaagtgtcggatgtagaggaaatggaaagtttgttctcagatggggacctgcttgattgcttcacaagaatgccagcttatgcggtaaacgcagaggaagatttagctgcaatcaggaaaacgcccgagatggatgtcggtcaagaagttaaagagcctgcaggagacagaaatcaatactcaaaccctgcagaaactttcctcaacaagctccacaggaaacacagtagggaggtgaaacaccaggccgcaaagaaagctaaacgcctagctgaaatccaggagtcaatgagagctgaaggtgatgccgaaccaaatgaaataagcgggacgatgggggcaatacccagcaacgccgaacttcctggcacgaatgatgccagacaagaactcacactcccaaccactaaacctgtccctgcaaggtgggaagatgcttcattcacagattctagtgtggaagaggagcaggttaaactccttggaaaagaaaccgttgaaacagcgacgcaacaagtcatcgaaggacttccttggaaacactggattcctcaattaaatgctgttggattcaaggcgctggaaattcagagggataggagtggaacaatgatcatgcccatcacagaaatggtctccgggctggaaaaagaggacttccctgaaggaactccaaaagagttggcacgagaattgttcgctatgaacagaagccctgccaccatccctttggacctgcttagagccagagactacggcagtgatgtaaagaacaagagaattggtgccatcacaaagacacaggcaacgagttggggcgaatacttgacaggaaagatagaaagcttaactgagaggaaagttgcgacttgtgtcattcatggagctggaggttctggaaaaagtcatgccatccagaaggcattgagagaaattggcaagggctcggacatcactgtagtcctgccgaccaatgaactgcggctagattggagtaagaaagtgcctaacactgagccctatatgttcaagacctctgaaaaggcgttaattgggggaacaggcagcatagtcatctttgacgattactcaaaacttcctcccggttacatagaagccttagtctgtttctactctaaaatcaagctaatcattctaacaggagatagcagacaaagcgtctaccatgaaactgctgaggacgcctccatcaggcatttgggaccagcaacagagtacttctcaaaatactgccgatactatctcaatgccacacaccgcaacaagaaagatcttgcgaacatgcttggtgtctacagtgagagaacgggagtcaccgaaatcagcatgagcgccgagttcttagaaggaatcccaactttggtaccctcggatgagaagagaaagctgtacatgggcaccgggaggaatgacacgttcacatacgctggatgccaggggctaactaagccgaaggtacaaatagtgttggaccacaacacccaagtgtgtagcgcgaatgtgatgtacacggcactttctagagccaccgataggattcacttcgtgaacacaagtgcaaattcctctgccttctgggaaaagttggacagcaccccttacctcaagactttcctatcagtggtgagagaacaagcactcagggagtacgagccggcagaggcagagccaattcaagagcctgagccccagacacacatgtgtgtcgagaatgaggagtccgtgctagaagagtacaaagaggaactcttggaaaagtttgacagagagatccactctgaatcccatggtcattcaaactgtgtccaaactgaagacacaaccattcagttgttttcgcatcaacaagcaaaagatgagaccctcctctgggcgactatagatgcgcggctcaagaccagcaatcaagaaacaaacttccgagaattcctgagcaagaaggacattggggacgttctgtttttaaactaccaaaaagctatgggtttacccaaagagcgtattcctttttcccaagaggtctgggaagcttgtgcccacgaagtacaaagcaagtacctcagcaagtcaaagtgcaacttgatcaatgggactgtgagacagagcccagacttcgatgaaaataagattatggtattcctcaagtcgcagtgggtcacaaaggtggaaaaactaggtctacccaagattaagccaggtcaaaccatagcagccttttaccagcagactgtgatgctttttggaactatggctaggtacatgcgatggttcagacaggctttccagccaaaagaagtcttcataaactgtgagaccacgccagatgacatgtctgcatgggccttgaacaactggaatttcagcagacctagcttggctaatgactacacagctttcgaccagtctcaggatggagccatgttgcaatttgaggtgctcaaagccaaacaccactgcataccagaggaaatcattcaggcatacatagatattaagactaatgcacagattttcctaggcacgttatcaattatgcgcctgactggtgaaggtcccacttttgatgcaaacactgagtgcaacatagcttacacccatacaaagtttgacatcccagccggaactgctcaagtttatgcaggagacgactccgcactggactgtgttccagaagtgaagcatagtttccacaggcttgaggacaaattactcctaaagtcaaagcctgtaatcacgcagcaaaagaagggcagttggcctgagttttgtggttggctgatcacaccaaaaggggtgatgaaagacccaattaagctccatgttagcttaaaattggctgaagctaagggtgaactcaagaaatgtcaagattcctatgaaattgatctgagttatgcctatgaccacaaggactctctgcatgacttgttcgatgagaaacagtgtcaggcacacacactcacttgcagaacactaatcaagtcagggagaggcactgtctcactttcccgcctcagaaactttctttaaccgttaagttaccttagagatttgaataagatggatattctcatcagtagtttgaaaagtttaggttattctaggacttccaaatctttagattcaggacctttggtagtacatgcagtagccggagccggtaagtccacagccctaaggaagttgatcctcagacacccaacattcaccgtgcatacactcggtgtccctgacaaggtgagtatcagaactagaggcatacagaagccaggacctattcctgagggcaacttcgcaatcctcgatgagtatactttggacaacaccacaaggaactctaaccaggcactttttgctgacccttatcaggcaccggagtttagcctagagccccacttctacttggaaacatcatttcgagttccgaggaaagtggcagatttgatagctggctgtggcttcgatttcgagaccaactcaccggaagaagggcacttagagatcactggcatattcaaagggcccctactcggaaaggtgatagccattgatgaggagtctgagacaacactgtccaggcatggtgttgagtttgttaagccctgccaagtgacgggacttgagttcaaagtagtcactattgtgtctgccgcaccaatagaggaaattggccagtccacagctttctacaacgctatcaccaggtcaaagggattgacatatgtccgcgcagggccataggctgaccgctccggtcaattctgaaaaagtgtacatagtattaggtctatcatttgctttagtttcaattacctttctgctttctagaaatagcttaccccacgtcggtgacaacattcacagcttgccacacggaggagcttacagagacggcaccaaagcaatcttgtacaactccccaaatctagggtcacgagtgagtctacacaacggaaagaacgcagcatttgctgccgttttgctactgactttgctgatctatggaagtaaatacatatctcaacgcaatcatacttgtgcttgtggtaacaatcatagcagtcattagcacttccttagtgaggactgaaccttgtgtcatcaagattactggggaatcaatcacagtgttggcttgcaaactagatgcagaaaccataagggccattgccgatctcaagccactctccgttgaacggttaagtttccattgatactcgaaagaGGTCAGCACCAGCTAGCCCGGGATGGATAAGAAGTACTCTATCGGACTCGATATCGGAACTAACTCTGTGGGATGGGCTGTGATCACCGATGAGTACAAGGTGCCATCTAAGAAGTTCAAGGTTCTCGGAAACACCGATAGGCACTCTATCAAGAAAAACCTTATCGGTGCTCTCCTCTTCGATTCTGGTGAAACTGCTGAGGCTACCAGACTCAAGAGAACCGCTAGAAGAAGGTACACCAGAAGAAAGAACAGGATCTGCTACCTCCAAGAGATCTTCTCTAACGAGATGGCTAAAGTGGATGATTCATTCTTCCACAGGCTCGAAGAGTCATTCCTCGTGGAAGAAGATAAGAAGCACGAGAGGCACCCTATCTTCGGAAACATCGTTGATGAGGTGGCATACCACGAGAAGTACCCTACTATCTACCACCTCAGAAAGAAGCTCGTTGATTCTACTGATAAGGCTGATCTCAGGCTCATCTACCTCGCTCTCGCTCACATGATCAAGTTCAGAGGACACTTCCTCATCGAGGGTGATCTCAACCCTGATAACTCTGATGTGGATAAGTTGTTCATCCAGCTCGTGCAGACCTACAACCAGCTTTTCGAAGAGAACCCTATCAACGCTTCAGGTGTGGATGCTAAGGCTATCCTCTCTGCTAGGCTCTCTAAGTCAAGAAGGCTTGAGAACCTCATTGCTCAGCTCCCTGGTGAGAAGAAGAACGGACTTTTCGGAAACTTGATCGCTCTCTCTCTCGGACTCACCCCTAACTTCAAGTCTAACTTCGATCTCGCTGAGGATGCAAAGCTCCAGCTCTCAAAGGATACCTACGATGATGATCTCGATAACCTCCTCGCTCAGATCGGAGATCAGTACGCTGATTTGTTCCTCGCTGCTAAGAACCTCTCTGATGCTATCCTCCTCAGTGATATCCTCAGAGTGAACACCGAGATCACCAAGGCTCCACTCTCAGCTTCTATGATCAAGAGATACGATGAGCACCACCAGGATCTCACACTTCTCAAGGCTCTTGTTAGACAGCAGCTCCCAGAGAAGTACAAAGAGATTTTCTTCGATCAGTCTAAGAACGGATACGCTGGTTACATCGATGGTGGTGCATCTCAAGAAGAGTTCTACAAGTTCATCAAGCCTATCCTCGAGAAGATGGATGGAACCGAGGAACTCCTCGTGAAGCTCAATAGAGAGGATCTTCTCAGAAAGCAGAGGACCTTCGATAACGGATCTATCCCTCATCAGATCCACCTCGGAGAGTTGCACGCTATCCTTAGAAGGCAAGAGGATTTCTACCCATTCCTCAAGGATAACAGGGAAAAGATTGAGAAGATTCTCACCTTCAGAATCCCTTACTACGTGGGACCTCTCGCTAGAGGAAACTCAAGATTCGCTTGGATGACCAGAAAGTCTGAGGAAACCATCACCCCTTGGAACTTCGAAGAGGTGGTGGATAAGGGTGCTAGTGCTCAGTCTTTCATCGAGAGGATGACCAACTTCGATAAGAACCTTCCAAACGAGAAGGTGCTCCCTAAGCACTCTTTGCTCTACGAGTACTTCACCGTGTACAACGAGTTGACCAAGGTTAAGTACGTGACCGAGGGAATGAGGAAGCCTGCTTTTTTGTCAGGTGAGCAAAAGAAGGCTATCGTTGATCTCTTGTTCAAGACCAACAGAAAGGTGACCGTGAAGCAGCTCAAAGAGGATTACTTCAAGAAAATCGAGTGCTTCGATTCAGTTGAGATTTCTGGTGTTGAGGATAGGTTCAACGCATCTCTCGGAACCTACCACGATCTCCTCAAGATCATTAAGGATAAGGATTTCTTGGATAACGAGGAAAACGAGGATATCTTGGAGGATATCGTTCTTACCCTCACCCTCTTTGAAGATAGAGAGATGATTGAAGAAAGGCTCAAGACCTACGCTCATCTCTTCGATGATAAGGTGATGAAGCAGTTGAAGAGAAGAAGATACACTGGTTGGGGAAGGCTCTCAAGAAAGCTCATTAACGGAATCAGGGATAAGCAGTCTGGAAAGACAATCCTTGATTTCCTCAAGTCTGATGGATTCGCTAACAGAAACTTCATGCAGCTCATCCACGATGATTCTCTCACCTTTAAAGAGGATATCCAGAAGGCTCAGGTTTCAGGACAGGGTGATAGTCTCCATGAGCATATCGCTAACCTCGCTGGATCTCCTGCAATCAAGAAGGGAATCCTCCAGACTGTGAAGGTTGTGGATGAGTTGGTGAAGGTGATGGGAAGGCATAAGCCTGAGAACATCGTGATCGAAATGGCTAGAGAGAACCAGACCACTCAGAAGGGACAGAAGAACTCTAGGGAAAGGATGAAGAGGATCGAGGAAGGTATCAAAGAGCTTGGATCTCAGATCCTCAAAGAGCACCCTGTTGAGAACACTCAGCTCCAGAATGAGAAGCTCTACCTCTACTACCTCCAGAACGGAAGGGATATGTATGTGGATCAAGAGTTGGATATCAACAGGCTCTCTGATTACGATGTTGATCATATCGTGCCACAGTCATTCTTGAAGGATGATTCTATCGATAACAAGGTGCTCACCAGGTCTGATAAGAACAGGGGTAAGAGTGATAACGTGCCAAGTGAAGAGGTTGTGAAGAAAATGAAGAACTATTGGAGGCAGCTCCTCAACGCTAAGCTCATCACTCAGAGAAAGTTCGATAACTTGACTAAGGCTGAGAGGGGAGGACTCTCTGAATTGGATAAGGCAGGATTCATCAAGAGGCAGCTTGTGGAAACCAGGCAGATCACTAAGCACGTTGCACAGATCCTCGATTCTAGGATGAACACCAAGTACGATGAGAACGATAAGTTGATCAGGGAAGTGAAGGTTATCACCCTCAAGTCAAAGCTCGTGTCTGATTTCAGAAAGGATTTCCAATTCTACAAGGTGAGGGAAATCAACAACTACCACCACGCTCACGATGCTTACCTTAACGCTGTTGTTGGAACCGCTCTCATCAAGAAGTATCCTAAGCTCGAGTCAGAGTTCGTGTACGGTGATTACAAGGTGTACGATGTGAGGAAGATGATCGCTAAGTCTGAGCAAGAGATCGGAAAGGCTACCGCTAAGTATTTCTTCTACTCTAACATCATGAATTTCTTCAAGACCGAGATTACCCTCGCTAACGGTGAGATCAGAAAGAGGCCACTCATCGAGACAAACGGTGAAACAGGTGAGATCGTGTGGGATAAGGGAAGGGATTTCGCTACCGTTAGAAAGGTGCTCTCTATGCCACAGGTGAACATCGTTAAGAAAACCGAGGTGCAGACCGGTGGATTCTCTAAAGAGTCTATCCTCCCTAAGAGGAACTCTGATAAGCTCATTGCTAGGAAGAAGGATTGGGACCCTAAGAAATACGGTGGTTTCGATTCTCCTACCGTGGCTTACTCTGTTCTCGTTGTGGCTAAGGTTGAGAAGGGAAAGAGTAAGAAGCTCAAGTCTGTTAAGGAACTTCTCGGAATCACTATCATGGAAAGGTCATCTTTCGAGAAGAACCCAATCGATTTCCTCGAGGCTAAGGGATACAAAGAGGTTAAGAAGGATCTCATCATCAAGCTCCCAAAGTACTCACTCTTCGAACTCGAGAACGGTAGAAAGAGGATGCTCGCTTCTGCTGGTGAGCTTCAAAAGGGAAACGAGCTTGCTCTCCCATCTAAGTACGTTAACTTTCTTTACCTCGCTTCTCACTACGAGAAGTTGAAGGGATCTCCAGAAGATAACGAGCAGAAGCAACTTTTCGTTGAGCAGCACAAGCACTACTTGGATGAGATCATCGAGCAGATCTCTGAGTTCTCTAAAAGGGTGATCCTCGCTGATGCAAACCTCGATAAGGTGTTGTCTGCTTACAACAAGCACAGAGATAAGCCTATCAGGGAACAGGCAGAGAACATCATCCATCTCTTCACCCTTACCAACCTCGGTGCTCCTGCTGCTTTCAAGTACTTCGATACAACCATCGATAGGAAGAGATACACCTCTACCAAAGAAGTGCTCGATGCTACCCTCATCCATCAGTCTATCACTGGACTCTACGAGACTAGGATCGATCTCTCACAGCTCGGTGGTGATtcaagggctgatcctaagaagaagaggaaggtttgaACGCGTGAATTGAGGGTACCCGAGTAGTTTTAGAGCTAGAAATAGCAAGTTAAAATAAGGCTAGTCCGTTATCAACTTGAAAAAGTGGCACCGAGTCGGTGCTTTTTTTCTAGACCCAGTCGACcgccgatGAACGGTTAAGTTTCCATTGATACTCGAAAGatgtcagcaccagctagcacaacacagcccatagggtcaactacctcaactaccacaaaaactgcaggcgcaactcctgccacagcttcaggcctgttcaccatcccggatggggatttctttagtacagcccgtgccatagtagccagcaatgctgtcgcaacaaatgaggacctcagcaagattgaggctatttggaaggacatgaaggtgcccacagacactatggcacaggctgcttgggacttagtcagacactgtgctgatgtaggatcatccgctcaaacagaaatgatagatacaggtccctattccaacggcatcagcagagctagactggcagcagcaattaaagaggtgtgcacacttaggcaattttgcatgaagtatgctccagtggtatggaactggatgttaactaacaacagtccacctgctaactggcaagcacaaggtttcaagcctgagcacaaattcgctgcattcgacttcttcaatggagtcaccaacccagctgccatcatgcccaaagaggggctcatccggccaccgtctgaagctgaaatgaatgctgcccaaactgctgcctttgtgaagattacaaaggccagggcacaatccaacgactttgccagcctagatgcagctgtcactcgaggtcgtatcactggaacaacaaccgctgaggctgttgtcactctaccaccaccataaCTACGTCTACATAACCGACGCCTACCCCAGTTTCATAGTATTTTCTGGTTTGATTGTATGAATAATATAAATAAAAAAAAAAAAAAAAAAAAAAAACTAGTggtACCGAGCTCGATCGTTCAAACATTTGGCAATAAAGTTTCTTAAGATTGAATCCTGTTGCCGGTCTTGCGATGATTATCATATAATTTCTGTTGAATTACGTTAAGCATGTAATAATTAACATGTAATGCATGACGTTATTTATGAGATGGGTTTTTATGATTAGAGTCCCGCAATTATACATTTAATACGCGATAGAAAACAAAATATAGCGCGCAAACTAGGATAAATTATCGCGCGCGGTGTCATCTATGTTACTAGATCGAATTCACTGGCCGTCGTTTAAACTATCAGTGTTTGACAGGATATATTGGCGGGTAAACCTAAGAGAAAAGAGCGTTTA

Fig. S6 continued

PVX cDNA

*RdRp*

35S promoter

*TGB*

*SpCas9*

Fig. S6 continued

*NbTOM1*_target sequence

sgRNA_scaffold

NLS

CP

Fig. S6 continued

nosT

RB

**Fig. S6 | Nucleotide sequence of pPZPVX-Cas9_NbTOM1.**

LB: Left-border sequence.

35S promoter: Promoter region of Cauliflower mosaic virus 35S RNA.

*RdRp*, *TGB* and *CP*: PVX genes.

*SpCas9*: Codon-optimized *SpCas9* gene from pDeCas9 (Fauser et al., 2014).

NLS: SV40-derived nuclear localization signal.

sgRNA_scaffold: sgRNA scaffold sequence for SpCas9 from pUC_AtU6-sgRNA (Endo et al., 2019).

nosT: Terminator region of *Agrobacterium tumefaciens* nopaline synthase gene.

RB: Right-border sequence.

>pPZPVX-AID_NbTOM1

LB

tggcaggatatattgtggtgtaaacaaattgacgcttagacaacttaataacacattgcggacgtttttaatgtactgaattaacgccgaattgctctagcattcgccattcaggctgcgcaactgttgggaagggcgatcggtgcgggcctcttcgctattacgccagctggcgaaagggggatgtgctgcaaggcgattaagttgggtaacgccagggttttcccagtcacgacgttgtaaaacgacggccagtgccaagctggcgcgtctcagaagaccaaagggcaatTGAGACTTTTCAACAAAGGGTAATATCCGGAAACCTCCTCGGATTCCATTGCCCAGCTATCTGTCACTTTATTGTGAAGATAGTGGAAAAGGAAGGTGGCTCCTACAAATGCCATCATTGCGATAAAGGAAAGGCCATCGTTGAAGATGCCTCTGCCGACAGTGGTCCCAAAGATGGACCCCCACCCACGAGGAGCATCGTGGAAAAAGAAGACGTTCCAACCACGTCTTCAAAGCAAGTGGATTGATGTGATATCTCCACTGACGTAAGGGATGACGCACAATCCCACTATCCTTCGCAAGACCCTTCCTCTATATAAGGAAGTTCATTTCATTTGGAGAGGAgaaaactaaaccatacaccaccaacacaaccaaacccaccacgcccaattgttacacacccgcttgaaaaagaaagtttaacaaatggccaaggtgcgcgaggtttaccaatcttttacagactccaccacaaaaactctcatccaagatgaggcttatagaaacattcgccccatcatggaaaaacacaaactagctaacccttacgctcaaacggttgaagcggctaatgatctagaggggttcggcatagccaccaatccctatagcattgaattgcatacacatgcagccgctaagaccatagagaataaacttctagaggtgcttggttccatcctaccacaagaacctgttacatttatgtttcttaaacccagaaagctaaactacatgagaagaaacccgcggatcaaggacattttccaaaatgttgccattgaaccaagagacgtagccaggtaccccaaggaaacaataattgacaaactcacagagatcacaacggaaacagcatacattagtgacactctgcacttcttggatccgagctacatagtggagacattccaaaactgcccaaaattgcaaacattgtatgcgaccttagttctccccgttgaggcagcctttaaaatggaaagcactcacccgaacatatacagcctcaaatacttcggagatggtttccagtatataccaggcaaccatggtggcggggcataccatcatgaattcgctcatctacaatggctcaaagtgggaaagatcaagtggagggaccccaaggatagctttctcggacatctcaattacacgactgagcaggttgagatgcacacagtgacagtacagttgcaggaatcgttcgcggcaaaccacttgtactgcatcaggagaggagacttgctcacaccggaggtgcgcactttcggccaacctgacaggtacgtgattccaccacagatcttcctcccaaaagttcacaactgcaagaagccgattctcaagaaaactatgatgcagctcttcttgtatgttaggacagtcaaggtcgcaaaaaattgtgacatttttgccaaagtcagacaattaattaaatcatctgacttggacaaatactctgctgtggaactggtttacttagtaagctacatggagttccttgccgatttacaagctaccacctgcttctcagacacactttctggtggcttgctaacaaagacccttgcaccggtgagggcttggatacaagagaaaaagatgcagctgtttggtcttgaggactacgcgaagttagtcaaagcagttgatttccacccggtggatttttctttcaaagtggaaacttgggacttcagattccaccccttgcaagcgtggaaagccttccgaccaagggaagtgtcggatgtagaggaaatggaaagtttgttctcagatggggacctgcttgattgcttcacaagaatgccagcttatgcggtaaacgcagaggaagatttagctgcaatcaggaaaacgcccgagatggatgtcggtcaagaagttaaagagcctgcaggagacagaaatcaatactcaaaccctgcagaaactttcctcaacaagctccacaggaaacacagtagggaggtgaaacaccaggccgcaaagaaagctaaacgcctagctgaaatccaggagtcaatgagagctgaaggtgatgccgaaccaaatgaaataagcgggacgatgggggcaatacccagcaacgccgaacttcctggcacgaatgatgccagacaagaactcacactcccaaccactaaacctgtccctgcaaggtgggaagatgcttcattcacagattctagtgtggaagaggagcaggttaaactccttggaaaagaaaccgttgaaacagcgacgcaacaagtcatcgaaggacttccttggaaacactggattcctcaattaaatgctgttggattcaaggcgctggaaattcagagggataggagtggaacaatgatcatgcccatcacagaaatggtctccgggctggaaaaagaggacttccctgaaggaactccaaaagagttggcacgagaattgttcgctatgaacagaagccctgccaccatccctttggacctgcttagagccagagactacggcagtgatgtaaagaacaagagaattggtgccatcacaaagacacaggcaacgagttggggcgaatacttgacaggaaagatagaaagcttaactgagaggaaagttgcgacttgtgtcattcatggagctggaggttctggaaaaagtcatgccatccagaaggcattgagagaaattggcaagggctcggacatcactgtagtcctgccgaccaatgaactgcggctagattggagtaagaaagtgcctaacactgagccctatatgttcaagacctctgaaaaggcgttaattgggggaacaggcagcatagtcatctttgacgattactcaaaacttcctcccggttacatagaagccttagtctgtttctactctaaaatcaagctaatcattctaacaggagatagcagacaaagcgtctaccatgaaactgctgaggacgcctccatcaggcatttgggaccagcaacagagtacttctcaaaatactgccgatactatctcaatgccacacaccgcaacaagaaagatcttgcgaacatgcttggtgtctacagtgagagaacgggagtcaccgaaatcagcatgagcgccgagttcttagaaggaatcccaactttggtaccctcggatgagaagagaaagctgtacatgggcaccgggaggaatgacacgttcacatacgctggatgccaggggctaactaagccgaaggtacaaatagtgttggaccacaacacccaagtgtgtagcgcgaatgtgatgtacacggcactttctagagccaccgataggattcacttcgtgaacacaagtgcaaattcctctgccttctgggaaaagttggacagcaccccttacctcaagactttcctatcagtggtgagagaacaagcactcagggagtacgagccggcagaggcagagccaattcaagagcctgagccccagacacacatgtgtgtcgagaatgaggagtccgtgctagaagagtacaaagaggaactcttggaaaagtttgacagagagatccactctgaatcccatggtcattcaaactgtgtccaaactgaagacacaaccattcagttgttttcgcatcaacaagcaaaagatgagaccctcctctgggcgactatagatgcgcggctcaagaccagcaatcaagaaacaaacttccgagaattcctgagcaagaaggacattggggacgttctgtttttaaactaccaaaaagctatgggtttacccaaagagcgtattcctttttcccaagaggtctgggaagcttgtgcccacgaagtacaaagcaagtacctcagcaagtcaaagtgcaacttgatcaatgggactgtgagacagagcccagacttcgatgaaaataagattatggtattcctcaagtcgcagtgggtcacaaaggtggaaaaactaggtctacccaagattaagccaggtcaaaccatagcagccttttaccagcagactgtgatgctttttggaactatggctaggtacatgcgatggttcagacaggctttccagccaaaagaagtcttcataaactgtgagaccacgccagatgacatgtctgcatgggccttgaacaactggaatttcagcagacctagcttggctaatgactacacagctttcgaccagtctcaggatggagccatgttgcaatttgaggtgctcaaagccaaacaccactgcataccagaggaaatcattcaggcatacatagatattaagactaatgcacagattttcctaggcacgttatcaattatgcgcctgactggtgaaggtcccacttttgatgcaaacactgagtgcaacatagcttacacccatacaaagtttgacatcccagccggaactgctcaagtttatgcaggagacgactccgcactggactgtgttccagaagtgaagcatagtttccacaggcttgaggacaaattactcctaaagtcaaagcctgtaatcacgcagcaaaagaagggcagttggcctgagttttgtggttggctgatcacaccaaaaggggtgatgaaagacccaattaagctccatgttagcttaaaattggctgaagctaagggtgaactcaagaaatgtcaagattcctatgaaattgatctgagttatgcctatgaccacaaggactctctgcatgacttgttcgatgagaaacagtgtcaggcacacacactcacttgcagaacactaatcaagtcagggagaggcactgtctcactttcccgcctcagaaactttctttaaccgttaagttaccttagagatttgaataagatggatattctcatcagtagtttgaaaagtttaggttattctaggacttccaaatctttagattcaggacctttggtagtacatgcagtagccggagccggtaagtccacagccctaaggaagttgatcctcagacacccaacattcaccgtgcatacactcggtgtccctgacaaggtgagtatcagaactagaggcatacagaagccaggacctattcctgagggcaacttcgcaatcctcgatgagtatactttggacaacaccacaaggaactctaaccaggcactttttgctgacccttatcaggcaccggagtttagcctagagccccacttctacttggaaacatcatttcgagttccgaggaaagtggcagatttgatagctggctgtggcttcgatttcgagaccaactcaccggaagaagggcacttagagatcactggcatattcaaagggcccctactcggaaaggtgatagccattgatgaggagtctgagacaacactgtccaggcatggtgttgagtttgttaagccctgccaagtgacgggacttgagttcaaagtagtcactattgtgtctgccgcaccaatagaggaaattggccagtccacagctttctacaacgctatcaccaggtcaaagggattgacatatgtccgcgcagggccataggctgaccgctccggtcaattctgaaaaagtgtacatagtattaggtctatcatttgctttagtttcaattacctttctgctttctagaaatagcttaccccacgtcggtgacaacattcacagcttgccacacggaggagcttacagagacggcaccaaagcaatcttgtacaactccccaaatctagggtcacgagtgagtctacacaacggaaagaacgcagcatttgctgccgttttgctactgactttgctgatctatggaagtaaatacatatctcaacgcaatcatacttgtgcttgtggtaacaatcatagcagtcattagcacttccttagtgaggactgaaccttgtgtcatcaagattactggggaatcaatcacagtgttggcttgcaaactagatgcagaaaccataagggccattgccgatctcaagccactctccgttgaacggttaagtttccattgatactcgaaagaGGTCAGCACCAGCTAGCCCGGGATGGATAAGAAGTACTCTATCGGACTCGCTATCGGAACTAACTCTGTGGGATGGGCTGTGATCACCGATGAGTACAAGGTGCCATCTAAGAAGTTCAAGGTTCTCGGAAACACCGATAGGCACTCTATCAAGAAAAACCTTATCGGTGCTCTCCTCTTCGATTCTGGTGAAACTGCTGAGGCTACCAGACTCAAGAGAACCGCTAGAAGAAGGTACACCAGAAGAAAGAACAGGATCTGCTACCTCCAAGAGATCTTCTCTAACGAGATGGCTAAAGTGGATGATTCATTCTTCCACAGGCTCGAAGAGTCATTCCTCGTGGAAGAAGATAAGAAGCACGAGAGGCACCCTATCTTCGGAAACATCGTTGATGAGGTGGCATACCACGAGAAGTACCCTACTATCTACCACCTCAGAAAGAAGCTCGTTGATTCTACTGATAAGGCTGATCTCAGGCTCATCTACCTCGCTCTCGCTCACATGATCAAGTTCAGAGGACACTTCCTCATCGAGGGTGATCTCAACCCTGATAACTCTGATGTGGATAAGTTGTTCATCCAGCTCGTGCAGACCTACAACCAGCTTTTCGAAGAGAACCCTATCAACGCTTCAGGTGTGGATGCTAAGGCTATCCTCTCTGCTAGGCTCTCTAAGTCAAGAAGGCTTGAGAACCTCATTGCTCAGCTCCCTGGTGAGAAGAAGAACGGACTTTTCGGAAACTTGATCGCTCTCTCTCTCGGACTCACCCCTAACTTCAAGTCTAACTTCGATCTCGCTGAGGATGCAAAGCTCCAGCTCTCAAAGGATACCTACGATGATGATCTCGATAACCTCCTCGCTCAGATCGGAGATCAGTACGCTGATTTGTTCCTCGCTGCTAAGAACCTCTCTGATGCTATCCTCCTCAGTGATATCCTCAGAGTGAACACCGAGATCACCAAGGCTCCACTCTCAGCTTCTATGATCAAGAGATACGATGAGCACCACCAGGATCTCACACTTCTCAAGGCTCTTGTTAGACAGCAGCTCCCAGAGAAGTACAAAGAGATTTTCTTCGATCAGTCTAAGAACGGATACGCTGGTTACATCGATGGTGGTGCATCTCAAGAAGAGTTCTACAAGTTCATCAAGCCTATCCTCGAGAAGATGGATGGAACCGAGGAACTCCTCGTGAAGCTCAATAGAGAGGATCTTCTCAGAAAGCAGAGGACCTTCGATAACGGATCTATCCCTCATCAGATCCACCTCGGAGAGTTGCACGCTATCCTTAGAAGGCAAGAGGATTTCTACCCATTCCTCAAGGATAACAGGGAAAAGATTGAGAAGATTCTCACCTTCAGAATCCCTTACTACGTGGGACCTCTCGCTAGAGGAAACTCAAGATTCGCTTGGATGACCAGAAAGTCTGAGGAAACCATCACCCCTTGGAACTTCGAAGAGGTGGTGGATAAGGGTGCTAGTGCTCAGTCTTTCATCGAGAGGATGACCAACTTCGATAAGAACCTTCCAAACGAGAAGGTGCTCCCTAAGCACTCTTTGCTCTACGAGTACTTCACCGTGTACAACGAGTTGACCAAGGTTAAGTACGTGACCGAGGGAATGAGGAAGCCTGCTTTTTTGTCAGGTGAGCAAAAGAAGGCTATCGTTGATCTCTTGTTCAAGACCAACAGAAAGGTGACCGTGAAGCAGCTCAAAGAGGATTACTTCAAGAAAATCGAGTGCTTCGATTCAGTTGAGATTTCTGGTGTTGAGGATAGGTTCAACGCATCTCTCGGAACCTACCACGATCTCCTCAAGATCATTAAGGATAAGGATTTCTTGGATAACGAGGAAAACGAGGATATCTTGGAGGATATCGTTCTTACCCTCACCCTCTTTGAAGATAGAGAGATGATTGAAGAAAGGCTCAAGACCTACGCTCATCTCTTCGATGATAAGGTGATGAAGCAGTTGAAGAGAAGAAGATACACTGGTTGGGGAAGGCTCTCAAGAAAGCTCATTAACGGAATCAGGGATAAGCAGTCTGGAAAGACAATCCTTGATTTCCTCAAGTCTGATGGATTCGCTAACAGAAACTTCATGCAGCTCATCCACGATGATTCTCTCACCTTTAAAGAGGATATCCAGAAGGCTCAGGTTTCAGGACAGGGTGATAGTCTCCATGAGCATATCGCTAACCTCGCTGGATCTCCTGCAATCAAGAAGGGAATCCTCCAGACTGTGAAGGTTGTGGATGAGTTGGTGAAGGTGATGGGAAGGCATAAGCCTGAGAACATCGTGATCGAAATGGCTAGAGAGAACCAGACCACTCAGAAGGGACAGAAGAACTCTAGGGAAAGGATGAAGAGGATCGAGGAAGGTATCAAAGAGCTTGGATCTCAGATCCTCAAAGAGCACCCTGTTGAGAACACTCAGCTCCAGAATGAGAAGCTCTACCTCTACTACCTCCAGAACGGAAGGGATATGTATGTGGATCAAGAGTTGGATATCAACAGGCTCTCTGATTACGATGTTGATCATATCGTGCCACAGTCATTCTTGAAGGATGATTCTATCGATAACAAGGTGCTCACCAGGTCTGATAAGAACAGGGGTAAGAGTGATAACGTGCCAAGTGAAGAGGTTGTGAAGAAAATGAAGAACTATTGGAGGCAGCTCCTCAACGCTAAGCTCATCACTCAGAGAAAGTTCGATAACTTGACTAAGGCTGAGAGGGGAGGACTCTCTGAATTGGATAAGGCAGGATTCATCAAGAGGCAGCTTGTGGAAACCAGGCAGATCACTAAGCACGTTGCACAGATCCTCGATTCTAGGATGAACACCAAGTACGATGAGAACGATAAGTTGATCAGGGAAGTGAAGGTTATCACCCTCAAGTCAAAGCTCGTGTCTGATTTCAGAAAGGATTTCCAATTCTACAAGGTGAGGGAAATCAACAACTACCACCACGCTCACGATGCTTACCTTAACGCTGTTGTTGGAACCGCTCTCATCAAGAAGTATCCTAAGCTCGAGTCAGAGTTCGTGTACGGTGATTACAAGGTGTACGATGTGAGGAAGATGATCGCTAAGTCTGAGCAAGAGATCGGAAAGGCTACCGCTAAGTATTTCTTCTACTCTAACATCATGAATTTCTTCAAGACCGAGATTACCCTCGCTAACGGTGAGATCAGAAAGAGGCCACTCATCGAGACAAACGGTGAAACAGGTGAGATCGTGTGGGATAAGGGAAGGGATTTCGCTACCGTTAGAAAGGTGCTCTCTATGCCACAGGTGAACATCGTTAAGAAAACCGAGGTGCAGACCGGTGGATTCTCTAAAGAGTCTATCCGGCCTAAGAGGAACTCTGATAAGCTCATTGCTAGGAAGAAGGATTGGGACCCTAAGAAATACGGTGGTTTCGTGTCTCCTACCGTGGCTTACTCTGTTCTCGTTGTGGCTAAGGTTGAGAAGGGAAAGAGTAAGAAGCTCAAGTCTGTTAAGGAACTTCTCGGAATCACTATCATGGAAAGGTCATCTTTCGAGAAGAACCCAATCGATTTCCTCGAGGCTAAGGGATACAAAGAGGTTAAGAAGGATCTCATCATCAAGCTCCCAAAGTACTCACTCTTCGAACTCGAGAACGGTAGAAAGAGGATGCTCGCTTCTGCTCGGTTTCTTCAAAAGGGAAACGAGCTTGCTCTCCCATCTAAGTACGTTAACTTTCTTTACCTCGCTTCTCACTACGAGAAGTTGAAGGGATCTCCAGAAGATAACGAGCAGAAGCAACTTTTCGTTGAGCAGCACAAGCACTACTTGGATGAGATCATCGAGCAGATCTCTGAGTTCTCTAAAAGGGTGATCCTCGCTGATGCAAACCTCGATAAGGTGTTGTCTGCTTACAACAAGCACAGAGATAAGCCTATCAGGGAACAGGCAGAGAACATCATCCATCTCTTCACCCTTACCAACCTCGGTGCTCCTCGAGCTTTCAAGTACTTCGATACAACCATCGATAGGAAGGCATACAGATCTACCAAAGAAGTGCTCGATGCTACCCTCATCCATCAGTCTATCACTGGACTCTACGAGACTAGGATCGATCTCTCACAGCTCGGTGGTGATtcaagggctgatcctaagaagaagaggaaggttggaggagggccaggagcagagtatgttagagcgttgtttgattttaacggtaacgatgaagaggatttaccctttaagaaaggcgacattctcaggattagggataaacctgaagagcaatggtggaatgctgaggatagtgaaggcaaacgaggaatgattttagtgccgtatgtggagaaatattcgggtgactacaaagatcatgatggtgattacaaagaccatgacatcgactacaaggatgatgatgataagtcagggATGACAGATGCTGAATATGTCAGAATCCACGAAAAGTTGGACATTTACACGTTTAAGAAGCAGTTCTTCAACAACAAGAAATCTGTTTCGCATAGGTGCTATGTGCTTTTCGAACTAAAACGTCGTGGAGAAAGACGGGCTTGCTTTTGGGGTTACGCGGTTAACAAACCACAATCAGGTACTGAACGAGGAATACACGCTGAAATCTTTTCTATCCGAAAGGTTGAGGAATATCTACGTGACAATCCTGGACAGTTCACTATCAATTGGTATTCTAGCTGGTCACCATGTGCAGATTGTGCTGAGAAGATTCTCGAATGGTACAATCAAGAGCTTAGAGGCAATGGACATACATTGAAAATATGGGCATGCAAGCTCTACTACGAAAAGAATGCCAGAAACCAAATTGGGCTTTGGAACTTGAGGGATAATGGAGTTGGGCTTAATGTCATGGTTTCTGAGCACTATCAATGTTGTCGGAAGATCTTCATACAAAGTTCCCATAACCAGTTGAATGAGAACAGATGGTTAGAGAAAACCCTTAAAAGAGCCGAGAAGAGAAGATCCGAACTGAGCATTATGATACAGGTCAAAATTCTGCATACCACTAAGAGTCCAGCTGTAacgcgtgactccggcggcagcATGACCAACCTGTCCGACATCATCGAGAAGGAGACGGGCAAGCAACTCGTGATCCAGGAGAGCATCCTCATGCTGCCAGAGGAGGTGGAGGAGGTCATCGGCAACAAGCCAGAGTCCGACATCCTGGTGCACACCGCCTACGACGAGTCCACCGACGAGAACGTCATGCTCCTGACCAGCGACGCCCCAGAGTACAAGCCATGGGCCCTCGTCATCCAGGACAGCAACGGGGAGAACAAGATCAAGATGCTGggtcctaagaagaaacgtaaagtagggccctgaACGCGTATACTCGGGTACCCTCAATTGTTTTAGAGCTAGAAATAGCAAGTTAAAATAAGGCTAGTCCGTTATCAACTTGAAAAAGTGGCACCGAGTCGGTGCTTTTTTTCTAGACCCAGTCGACcgccgatGAACGGTTAAGTTTCCATTGATACTCGAAAGatgtcagcaccagctagcacaacacagcccatagggtcaactacctcaactaccacaaaaactgcaggcgcaactcctgccacagcttcaggcctgttcaccatcccggatggggatttctttagtacagcccgtgccatagtagccagcaatgctgtcgcaacaaatgaggacctcagcaagattgaggctatttggaaggacatgaaggtgcccacagacactatggcacaggctgcttgggacttagtcagacactgtgctgatgtaggatcatccgctcaaacagaaatgatagatacaggtccctattccaacggcatcagcagagctagactggcagcagcaattaaagaggtgtgcacacttaggcaattttgcatgaagtatgctccagtggtatggaactggatgttaactaacaacagtccacctgctaactggcaagcacaaggtttcaagcctgagcacaaattcgctgcattcgacttcttcaatggagtcaccaacccagctgccatcatgcccaaagaggggctcatccggccaccgtctgaagctgaaatgaatgctgcccaaactgctgcctttgtgaagattacaaaggccagggcacaatccaacgactttgccagcctagatgcagctgtcactcgaggtcgtatcactggaacaacaaccgctgaggctgttgtcactctaccaccaccataaCTACGTCTACATAACCGACGCCTACCCCAGTTTCATAGTATTTTCTGGTTTGATTGTATGAATAATATAAATAAAAAAAAAAAAAAAAAAAAAAAACTAGTggtACCGAGCTCGATCGTTCAAACATTTGGCAATAAAGTTTCTTAAGATTGAATCCTGTTGCCGGTCTTGCGATGATTATCATATAATTTCTGTTGAATTACGTTAAGCATGTAATAATTAACATGTAATGCATGACGTTATTTATGAGATGGGTTTTTATGATTAGAGTCCCGCAATTATACATTTAATACGCGATAGAAAACAAAATATAGCGCGCAAACTAGGATAAATTATCGCGCGCGGTGTCATCTATGTTACTAGATCGAATTCACTGGCCGTCGTTTAAACTATCAGTGTTTGACAGGATATATTGGCGGGTAAACCTAAGAGAAAAGAGCGTTTA

Fig. S7 continued

*RdRp*

PVX cDNA

35S promoter

*TGB*

*nSpCas9-NG*

Fig. S7 continued

NLS

*PmCDA*

Fig. S7 continued

sgRNA_scaffold

*UGI*

*NbTOM1*_sgRNA

CP

CP

nosT

RB

**Fig. S7 | Nucleotide sequence of pPZPVX-AID_NbTOM1.**

LB: Left-border sequence.

35S promoter: Promoter region of Cauliflower mosaic virus 35S RNA.

*RdRp*, *TGB* and *CP*: PVX genes.

*nSpCas9-NG*: Nickase *SpCas9* gene from nSpCas9-NGv1-AID-UGI (Endo et al., 2019).

NLS: SV40-derived nuclear localization signal.

*PmCDA*: Codon-optimized Cytidine deaminase of *Petromyzon marinus* from nSpCas9-NGv1-AID-UGI (Endo et al., 2019).

*UGI*: *Uracil glycosylase inhibitor* from nSpCas9-NGv1-AID-UGI (Endo et al., 2019).

sgRNA_scaffold: sgRNA scaffold sequence for SpCas9 from pUC_AtU6-sgRNA (Endo et al., 2019).

nosT: Terminator region of *Agrobacterium tumefaciens* nopaline synthase gene.

RB: Right-border sequence.

**Supplementary Table S1 | Primers used in this study**

| for PVX construction | Sequence (5′ > 3′) | |  |
| --- | --- | --- | --- |
| MCS_oligo_5 | CTAGCCCGGGCGATCGCACGTGACGCGTTTGCAAG | |  |
| MCS_oligo_3 | TCGACTTGCAAACGCGTCACGTGCGATCGCCCGGG | |  |
| pPVX201_MluI_F | ataaACGCGTCTCAGAAGACCAAAGGGCAATTG | |  |
| pPVX201_PmeI_R | GTTTAAACGACGGCCAGTGAATTCGA | |  |
| SpCas9_XmaI_F | aaCCCGGGATGGATAAGAAGTACTCTAT | |  |
| SpCas9_MluI_R | ataaACGCGTTCAAACCTTCCTCTTCTTCTTAGGA | |  |
| Sp_sgRNA_MluI_F | acttACGCGT*****(sgRNA seq. 20 nt)***** | |  |
| Sp_sgRNA_SalI_R | ataaGTCGACTGGGTCTAGAAAAAAAGCACCGACT | |  |
| for sgRNA | Forward primer (5′ > 3′) | Reverse primer (5′ > 3′) |  |
| NbTOM1 | attgAATTGAGGGTACCCGAGTA | aaacTACTCGGGTACCCTCAATT |  |
| NbPDS | attgTGCGATGCCTAACAAGCCAG | aaacCTGGCTTGTTAGGCATCGCA |  |
| NbTOM1_AID | attgATACTCGGGTACCCTCAATT | aaacAATTGAGGGTACCCGAGTAT |  |
| for CAPS | Forward primer (5′ > 3′) | Reverse primer (5′ > 3′) | Restriction enzyme |
| NbTOM1s_CAPS | GACTGTATCCTCAAAGCTTTGATC | CCTTAGGATGGAAGAGAAACACT | AvaI |
| NbPDSa_CAPS | CTGCTAACTCGTATGGTGACTCATA | ATCAACGCTGAAGGGAGAAATTTAC | BstNI |
| for sequencing | Forward primer (5′ > 3′) | Reverse primer (5′ > 3′) |  |
| NbTOM1a | GTCAGGGTGCACGTGAACGCA | GGTCCGACATAATTGCTGTCCT |  |
| NbTOM1b | CGGTGCACGTGAACCCATGGT | CAGAAGTAATTGCCGTACTGCT |  |
| for characterization | Forward primer (5′ > 3′) | Reverse primer (5′ > 3′) |  |
| T-DNA check | CTCACCGGAAGAAGGGCACTTAGA | CCGAAGATAGGGTGCCTCTCG |  |
| PVX_RT-PCR | CAAGTGGAGGGACCCCAAGGATAGC | CGACACTTCCCTTGGTCGGAAGGCT |  |

**Supplementary Table S2 | Summary of three independent experiments for the mutation rates of regenerated shoots from leaves infiltrated with mixtures of *Agrobacterium* strains harboring pRI-p19, pDe-Cas9, and pPZP2028_AtU6Sp_NbTOM1.**

|  | No. of regenerated shoots | No. of shoots with mutations | Mutation rate^1^ | No. of  shoots with  T-DNA | T-DNA  integration rate^1^ |
| --- | --- | --- | --- | --- | --- |
| Experiment 1 | 21 | 0 | 1.56% | 2 | 7.8% |
| Experiment 2 | 21 | 0 |  | 0 |  |
| Experiment 3 | 22 | 1 |  | 3 |  |

^1^Mutation rate and T-DNA integration rate were calculated from the sum of three independent experiments.
